# Insights for the future of health system partnerships in low- and middle-income countries: a systematic literature review

**DOI:** 10.1186/s12913-020-05435-8

**Published:** 2020-06-22

**Authors:** Simone Fanelli, Fiorella Pia Salvatore, Gianluigi De Pascale, Nicola Faccilongo

**Affiliations:** 1 Department of Economics and Management, University of Parma, Via J. F. Kennedy, 6, Parma, Italy; 2 Department of Economics, University of Foggia, Foggia, Italy

**Keywords:** Public-private partnership, Low- middle-income countries, Systematic literature review, Healthcare management, Content analysis

## Abstract

**Background:**

Despite growing support for the private sector involvement in the provision of public health services in Low- and Middle-Income Countries (LMICs), a lack of clear information on the future of the provision of such services restricts the ability of managers and policy-makers to assess how feasible integration between public and private actors may be in these countries. This paper presents a systematic literature review which traces the dynamics and boundaries of public-private partnerships for the healthcare sector in LMICs.

**Methods:**

A total of 723 articles indexed in Scopus were initially submitted to bibliometric analysis. Finally, 148 articles published in several academic journals were selected for independent full-text review by two researchers. Content analysis was made in order to minimise mistakes in interpreting the findings of studies in the sample.

**Results:**

Public-private partnerships identified through the content analysis were categorised into four research areas: 1) Transfer of resources; 2) Co-production of health goods and services; 3) Governance networks; 4) Criteria for successful partnership development.

**Conclusions:**

The four main research areas supply suggestions for a future research agenda, and managerial and policy implications for partnerships in LMICs.

## Background

The late twentieth century was a time of decentralisation of public services and privatisation. Governments sold and rented national properties with the aim of ensuring effectiveness and efficiency in the distribution of public services. Guasch finds that intervention in the public sector by private businesses enhanced the efficiency of the entire production process [1]. Enforcing private investments in innovative sectors made it possible to obtain profits and consequently generated economic expansion. The trend towards structures being composed by public and private segments first appeared in the early 1990s [2, 3]. Villani et al. state that countries’ use of “mixed forms” is motivated both by the capabilities of the private sector and by national spending on innovation processes trying to meet the growing public debt [4].

According to most researchers, the involvement of the private sector in the provision of public services is a key factor for the wellness of populations, especially for those of Low- and Middle-Income Countries (LMICs) [5].

Over recent decades, different forms of partnership in public sectors have been developed [6]. Such partnerships have largely been successful in ensuring better provision of services in the healthcare sector. Nonetheless, there is a recurrent debate on the public-private role in providing services in LMICs [7]. Controversies between exponents of public and private systems gained momentum due to the economic collapse of the early 2000s [8]. Guidelines from the International Monetary Fund advocated complete involvement of the private sector in providing healthcare services with the aim of decreasing public debt [9]. The World Health Organization (WHO) also stated that a pragmatic path allowing the engagement of the private sector would be the best solution in developing countries [10]. On the other hand, Oxfam, an international organisation working for global poverty reduction through development projects, criticised this strategy and stressed the importance of increased public sector participation for general and fair access to healthcare. Other authors confirm that private forms of the market cannot provide public services, or health care [11]. The absence of full competition, problems of information asymmetry, and the presence of externalities are just few issues which can affect health market efficiency and its ability to maximize individual utilities [12].

To date, the intergovernmental collaboration system planned by WHO has given way to a disjointed healthcare governance system, identified as a set of “formal and informal institutions, norms and processes which govern or directly influence global health policy and outcomes” [13]. Today, firms, corporations, civil society and private philanthropists are all actors in Public-Private Partnerships (PPPs) and take part in joint public-private decision-making processes, on complex health problems ranging from vaccination programs to under-nutrition [14]. Barr identifies no common understanding of what exactly a PPP is, but international enthusiasm for use of the PPP model to improve healthcare [2]. Such enthusiasm was clear in the early 2000s. According to Ahn et al. “the challenges of the myriad unmet health needs of developing nations can begin to be fulfilled” by PPPs [15]. Buse found that the international health landscape reconfiguration was occurring thanks to the rapid spread of PPPs [16]. Nishtar on the other hand, considered PPP the only way of providing vaccines and new drugs to the poorest populations in response to the “global call for action” [17].

A clear and accurate definition of PPP has not as yet been agreed on. WHO finds that the term PPP covers a wide variety of ventures involving a diversity of arrangements, varying with regard to participants, legal status, governance, management, policy-setting prerogatives, contributions, and operational roles [18]. In large-scale discussion, there is a disconnection between those wishing for global public-based healthcare and those advocating private sector provision in fields where the public sector has failed. Governments have in fact increasingly relied on private sector participation for healthcare sector improvement [3]. Indeed, some private-sector supporters claim that by increasing the number of providers, PPPs can ensure better quality services at “optimal” cost [19]. Their structure enables them to cope with rising healthcare expenses and the decreasing public budgets at the same time [20]. This aspect is considered the main benefit of PPPs, since the public and private sectors acting separately are unable to solve many emerging national health questions [21].

An in-depth investigation of PPPs reveals strong growth in industrialised countries where they increase the utilisation of healthcare infrastructures such as technologies, medical devices, clinical and non-clinical services, and facility management services [22]. Public approval was also found in developing countries where health systems are predominantly “mixed” [23]. In many LMICs, the public health system exists in parallel with the non-public health system, where for-profit organisations play a key role [24]. In these cases, inadequate public resources and a poorly defined regulatory system in the private sector can weaken the efficiency of the healthcare system [24]. In most LMICs, low capacity to meet health needs is a result of insufficient drugs supply, poor healthcare infrastructures, scarce resources and generally low quality of care [25, 26]. For these reasons, private sector involvement in healthcare in LMICs can enable the most vulnerable citizens to have access to health care [27].

Because of their ability to generate efficiency and effectiveness, PPPs are the main tool used to implement public, health and social policies in LMICs [23]. They provide potential access to public services and ensure resources can be allocated in an effective and impartial way. PPPs appear to be “key structures” for the definition, evaluation and delivery of many healthcare services in LMICs. Statistically, many studies reveal that market failure occurs frequently in the provision of health services by the private sector [11, 12, 28]. Managers and policy-makers adopting PPPs therefore try to eliminate failures in such a way that private resources can be exploited for the common benefit [29]. They identify the private sector as a resource for improving healthcare coverage and quality of services, and aim to exploit funding and know-how, and at the same time, mitigate damage caused by unregulated private provision [30]. Roeder and Labrie find that a combination of public contributions, regulations and private healthcare provision can provide adequate health services [31]. However, this is only the case in certain countries, because this type of system requires there to be robust national institutions, condition not existing in all LMICs [29].

A systematic review of existing literature is necessary for adequate discussion of this topic. Reinforced evidence-based on the performance of the public and private health sectors is crucial to guide policy-makers towards the definition of proper policy [29]. However, health services are not regularly categorised between public and private providers and often actors are part of both public and private healthcare entities, so it is complex to map the exact involvement of public and private actors. In addition, the concept of equity is often believed to be inadequately addressed through PPP health projects, and since PPPs policies will continue to have a growing impact on the political agenda of LMICs, rigorous research is imperative for decision-makers. The sparse literature reviews on health PPPs of LMICs tend to focus on inequality and global health initiatives [32]. The present systematic literature review (SLR), on the other hand, attempts as its main purpose to meet the need to comprehend the dynamics and boundaries of PPPs for the healthcare sector in LMICs. Although SLR exist on fields such as models of public-private engagements for provision and financing of health programs for LMICs, to date, no SLR has aimed to identify research areas (RAs) to be addressed in future studies. Results of the SLR provide a useful overview of the phenomenon and a useful baseline for managerial and policy implications for the evolution of PPPs in LMIC healthcare sectors. Offering insights relating to future research needs, the SLR investigates the topic through a cross-sectoral viewpoint taking into account the managerial and policy implications of PPPs as the most widely used structure of partnership in national health systems.

In sum, this SLR addresses the following research questions: How is the literature on health system PPPs in developing countries evolving? What is the focus of the literature on health system PPPs in developing countries? What are the implications of the research?

## Methods

This study builds on an SLR. A literature review is a general definition of the activity of mapping and assessing the existing body of knowledge in order to find potential research gaps [33], while an SLR involves a procedure designed as an interactive cycle to perform the mapping and assessment.

The first step of the procedure was to design the research questions (see Background).

Recommendations for LMIC managers and policy-makers are outlined in the light of the results and described in the discussion. In addition, a protocol for data search and article selection was established. Firstly, the most common available scientific databases by focus area, and next those gathering items regarding socio-economic and related issues were identified. Analysing the existing literature on the differences between Scopus and Web of Science databases, Mishra et al. note that Scopus includes more than 20,000 peer-reviewed journals, and thus provides the widest coverage of academic journals [34]. Chughtai and Blanchet state that Web of Science includes over 12,000 peer-reviewed journals and almost all journals in Web of Science are included in Scopus [35]. On this basis, it was decided to use the Scopus database. We used the PRISMA flow diagram [36] to obtain the final set of articles to work on. PRISMA considers four different phases:

1. Identification – searching records on identified database;
2. Screening – to exclude duplicates by reading title and abstract;
3. Eligibility – reading full text;
4. Inclusion – records to process with analysis.

The following keywords were used: “*Public-Private Partnership*” OR “*ppp”* OR “project financing” AND “health*” OR “*hospital*” AND “*low-income*” OR “*middle-income”* OR “developing countr*” OR “emergent nation*” OR “*third world*” OR “underdeveloped nation*”.

**Fig. 1:**
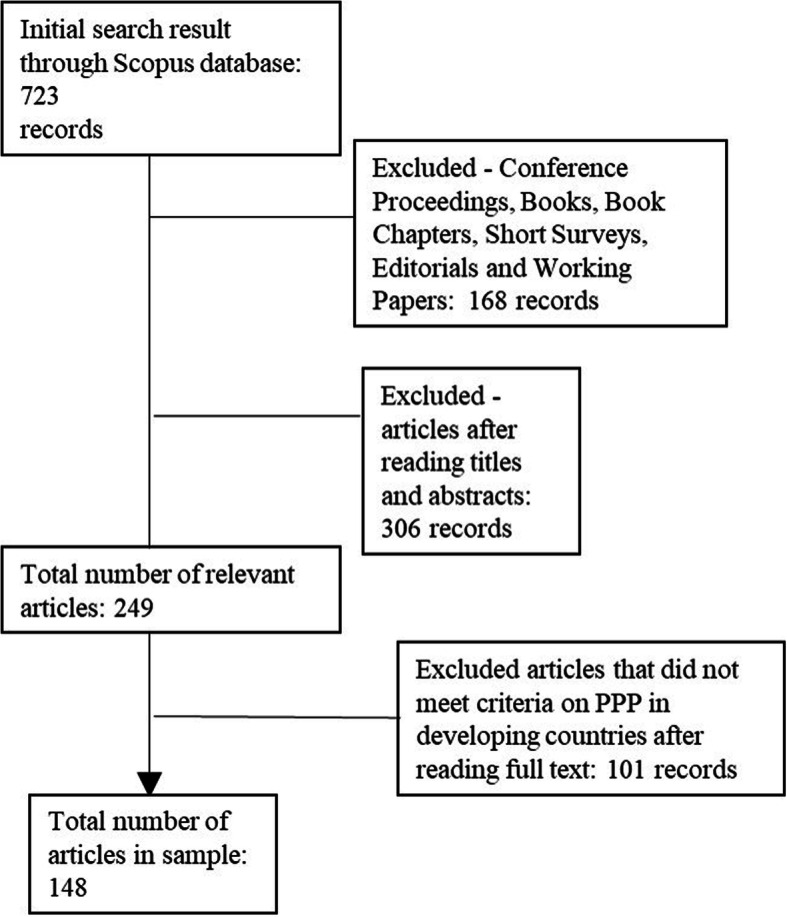
Workflow of data selection. The figure show the PRISMA flow diagram

The research period to 1990–to date was determined because research has noted that the term PPP rarely appeared in academic articles before 1990 [2]. The dataset was downloaded in January 2019 and yielded 723 items. As shown in Fig. 1, grey literature, was excluded.

Two researchers took part in finalising the set of articles for analysis. The inter-rater reliability [37], i.e. the rate of agreement between the choices made by the two judges with the calculation of the Cohen’s Kappa, was assessed. Cohen K was 0.8307.

The result was compared to the indicative cut-off, where values above 0.61 are deemed acceptable. The final dataset includes 148 peer-reviewed articles within the timeframe 1993–2018. The dataset comprises case studies and empirical studies (both qualitative and quali-quantitative studies). All these documents were used for descriptive and content analysis through bibliometric analysis.

### Descriptive analysis

Descriptive analysis was performed by reporting the evolution in time of the number of published articles, as well as their impact assessed by number of citations. For citations, we follow the work of Massaro et al. [38] and use the Citation per Year (CPY) index. This allow us to account for the lag time for citations that biases older articles compared to more recently published ones.

### Bibliometric analysis for content evaluation

As noted by Secundo et al. [39] and Christoffersen [40], content analysis is a method of examining research topics in order to deliver insights and critiques. VOSviewer, a software for visualising networks and clusters was used [41]. The Visualization of Similarities technique was developed by van Eck and Waltman [42]. It is based on the concept of distances between two objects, and is therefore linked to the basic assumptions underlying clustering techniques. VOSviewer considers the distances in different ways and was set up to display the results as described in the next paragraph.

Bibliographic coupling looks at a third publication cited by two articles in the sample. It concerns the overlapping literature between articles. The more the literature overlaps, the stronger the link between the considered articles in terms of RA. In order to include the most influential articles clustering with other articles, the coupling was set up to identify documents having at least six citations in common. Below this threshold, most of the articles did not group with other elements and were excluded by the software algorithm. The above analyses were both conducted using the fractional counting method devised by Leydesdorff and Opthof.

## Results

Results discern between descriptive and content analysis. Descriptive analysis includes: evolution in time, top 15 journals, top 10 authors. Content analysis concerns the identification of the RAs from the clusters emerging with the bibliographic coupling technique.

### Evolution in time, top authors and journals

**Fig. 2:**
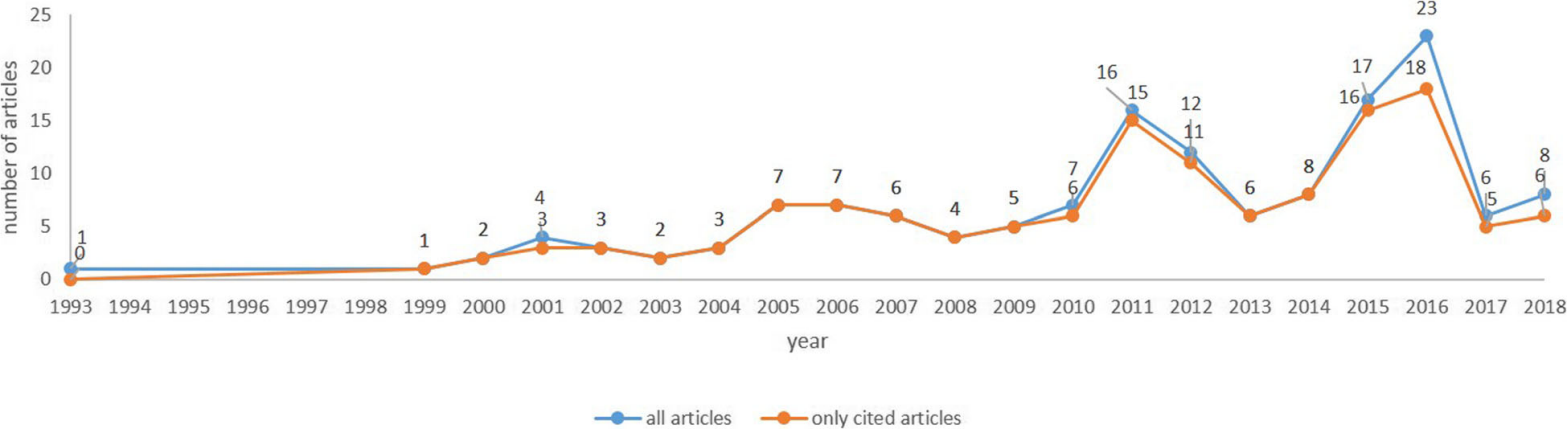
Trend of all articles versus cited articles. The graph shows the trend of academic articles (line blue) and the academic articled cited (line orange) in the period 1993–1998

**Fig. 3:**
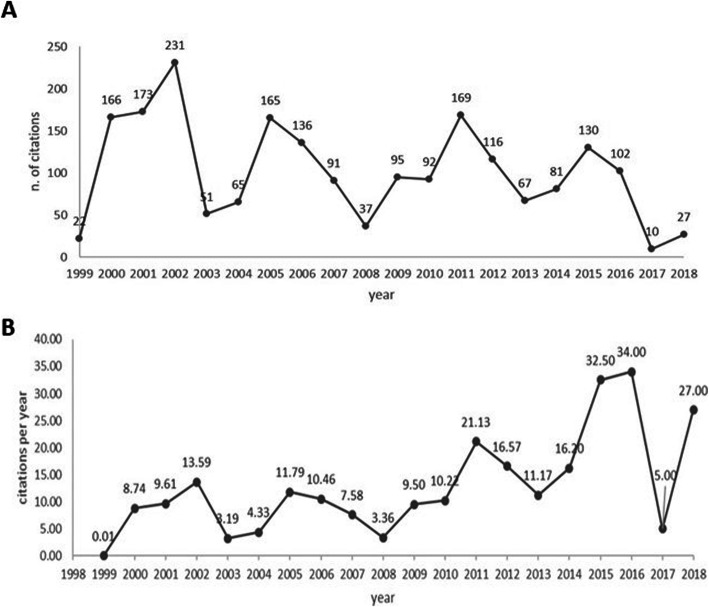
Number of citations and weighted citations per year. (A) The graph shows the number of citations per year in the period 1999–2018. (B) The graph shows the number of weighted citations per year, using the citation per-year index, in the period 1999–2018

Articles on PPP in the health sector were examined in the timeframe from 1993 through 2018 (Fig. 2 – blue line). From 1993 to 1999 only one item was written, but from 1998 on, attention increased and about 5 items per year were published until 2010. The first peak of 16 articles was seen in 2011, then 17 in 2015 and 23 in 2016. However, the data must be considered together with the orange line showing the number of citations the articles received over time. The joint reading of the two lines gives a preliminary indication of the influence of the articles. No citations were received by the first article in 1993, so the timeframe for citations becomes 1999–2018. The two lines however provide no information on the number of citations or the lag time, which are shown in Fig. 3.

Figure 3A shows the number of citations per year. It is noticeable that in the period 2000–2007, more than a thousand citations were received by 33 articles (Fig. 2). This trend is replicated in the periods 2009–2012 and 2014–2016 (Figs. 2 and 3A), but with some clear differences: the number of articles cited is higher (this means that each article may have lower impact than those in the period 2000–2007); the periods are shorter than the seven years of 2000–2007; the distribution over time of citations may mean that they are less influential in 2000–2007; the age of the articles is different; those published earlier might have had more chance to be read and cited. There is an issue linked to the lag time between articles published at different times, which may impact on the significance of their influence, even when the number of citations is significant.

Figure 3B attempts to resolve the issue of lag time by reporting the CPY [38, 39]. It shows the values of the CPY calculated considering the breadth of the timeframe. For this analysis, the focus moves to the two most recent periods as the influence score is higher than the first one from 2000 to 2007. Articles published in 2018 received 27 citations during the year of publication, which places those articles in third position in the overall rank of the CPY. However, a further aspect is the number of articles published, which affects the influence of each article and makes citations more difficult to interpret. Without taking this into account, the data presented in Figs. 3 are biased. To solve this problem, Table 1 reports the top 10 cited articles and related CPY.

**Table 1:** Top 10 articles ranked by CPY

Authors (Year)	Title	Source title	Cited by	CPY	Ranking CPY
Buse and Walt (2000)	Global public-private partnerships: Part II - What are the health issues for global governance?	Bull World Health Organ	161	8.94	2
Widdus (2001)	Public-private partnerships for health	Bull World Health Organ	134	7.88	4
Molyneux and Zagaria (2002)	Lymphatic filariasis elimination Progress in global programme development	Ann Trop Med Parasitol	128	8	3
Hotez and Ferris (2006)	The antipoverty vaccines	Vaccine	119	9.91	1
Gupta et al. (2002)	Increasing transparency in partnerships for health - Introducing the Green Light Committee	Trop Med Int Health	97	6.06	5
Bathurst and Hentschel (2006)	Medicines for Malaria Venture: sustaining antimalarial drug development	Trends Parasitol	59	4.92	6
Widdus (2005)	Public-private partnerships: An overview	Trans R Soc Trop Med Hyg	52	4	9
Lang and Greenwood (2003)	The development of lapdap, an affordable new treatment for malaria	Lancet Infect Dis	47	3.61	10
Mavalankar et al. (2009)	Saving mothers and newborns through an innovative partnership with private sector obstetricians: Chiranjeevi scheme of Gujarat, India	Int J Gynaecol Obstet	41	4.55	8
Zhang et al. (2010)	Control of neglected tropical diseases needs a long-term commitment	BMC Med	39	4.88	7

Following this rationale, it was found that the most influential articles by CPY are located in the earliest period 2000–2007. This kind of information is necessary to raise researcher awareness of potential problems in handling scientific archives. Moreover, the fact that these articles are at the top of the CPY ranking does not mean that they are included in the cluster analysis performed to assess the content of the articles. But the joint reading of the descriptive tables in Figs. 2 and 3 is an introduction to a the subsequent more detailed investigation using bibliographic coupling for bibliometric analysis.

As follow-up to Table 1, it was attempted to identify the most influential journals, and whether information from the column “source title” in the top ten articles by CPY is confirmed when considering cumulative citations of all articles published by the journals. The list below shows the top fifteen journals ranked by number of citations received by the articles published:

1. Bulletin of the World Health Organization (347 citations; 4 articles)
2. Tropical Medicine and International Health (199 citations; 5 articles)
3. Annals of Tropical Medicine and Parasitology (128 citations; 1 article)
4. Vaccine (119 citations; 1 article)
5. Health Affairs (112 citations; 9 articles)
6. Transactions of the Royal Society of Tropical Medicine and Hygiene (84 citations; 2 articles)
7. Health Research Policy and Systems (61 citations; 4 articles)
8. Trends in Parasitology (59 citations; 1 article)
9. Lancet Infectious Diseases (47 citations; 1 article)
10. Health Policy and Planning (46 citations; 4 articles)
11. International Journal of Gynecology and Obstetrics (41 citations; 1 article)
12. American Journal of Public Health (39 citations; 2 articles)
13. BMC Medicine (39 citations; 1 article)
14. Health Policy (38 citations; 2 articles)
15. Nature Reviews Clinical Oncology (37 citations; 1 article)

The *Bulletin of the World Health Organization* remains in top position with 4 items and 347 citations in all, but other journals, move up or down according to the number of articles and related citations computed in the new ranking.

### Content analysis: research areas

**Fig. 4:**
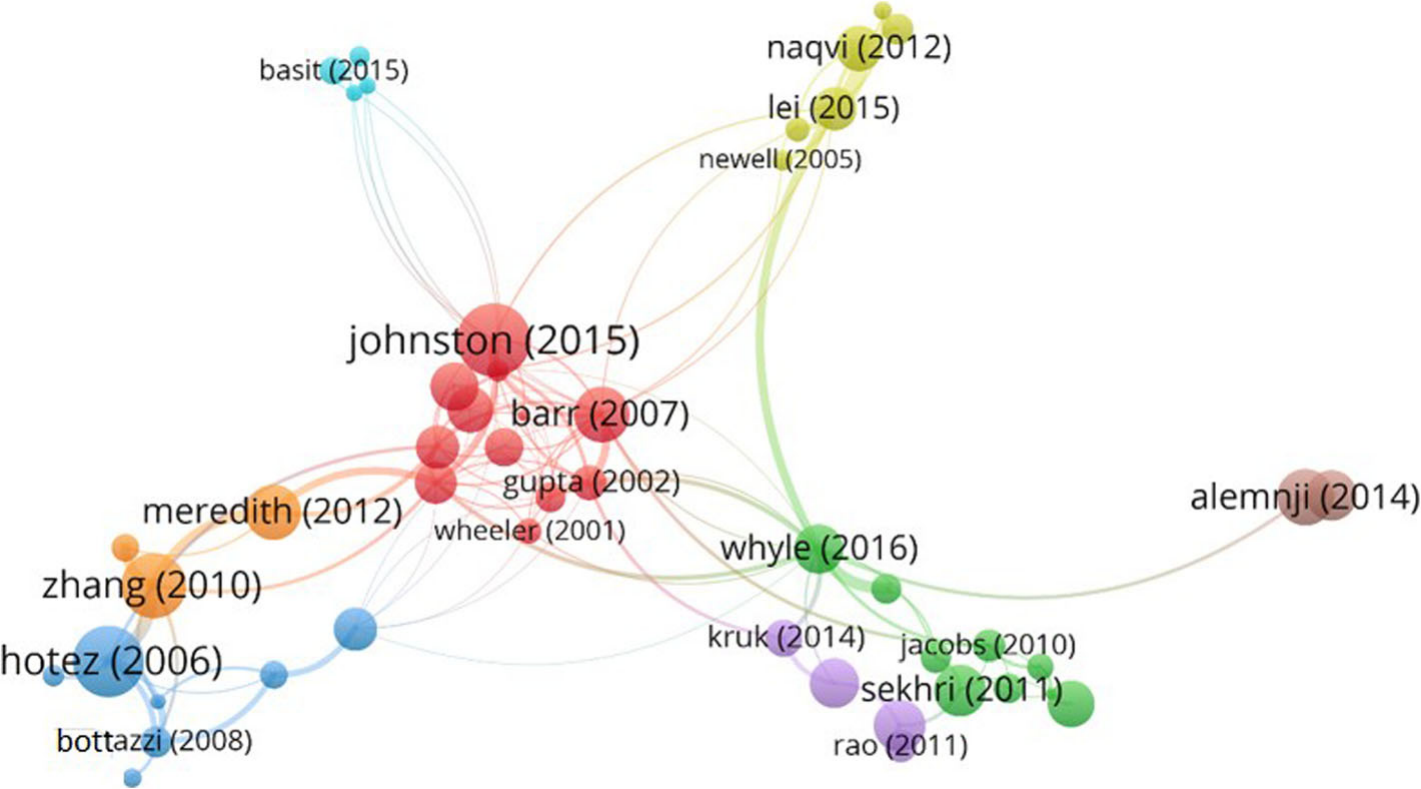
Clusters considering articles cited 6 times at least. In the network visualisation, authors of the articles considered are represented by their label and by a circle. The size of the label and the circle of each item is determined by the weight of the item itself: the higher the weight of an item, the larger the label and the circle of the item. The distance between two items indicates the relatedness of the items in terms of co-citation links. For some items, the label is not displayed. This is done in order to avoid overlapping labels. The colour of an item is determined by the cluster to which the item belongs. The strongest co-citation links between articles are also represented by lines

The 47 scientific articles selected through the bibliometric clustering process (Table 2 and Fig. 4) were read carefully by two researchers in order to identify the RAs. Most of the articles are qualitative studies (41 articles), but six studies use a quali-quantitative approach. Table 3 summarizes the main characteristics of these six articles.

**Table 2:** Clusters by bibliometric coupling of documents

Cluster*	Authors (Citations)
**Cluster 1 – red**	Anderson (6) [43]; Barr (36) [2]; Buse and Walt (161) [16]; Gupta et al. (97) [44]; Hein and Kohlmorgen (17) [45]; Johnston and Finegood (27) [5]; Lo (128) [46]; Molyneux and Zagaria (128) [47]; Peters and Phillips (37) [48]; Streefland (24) [49]; Vian et al. (22) [50]; Wheeler and Berkley (36) [51].
**Cluster 2 – green**	Ejaz et al. (20) [52]; Holden (20) [25]; Jacobs et al. (15) [53]; La Forgia and Harding (20) [54]; McIntosh et al. (8) [30]; Palmer and Mills (20) [55]; Palmer (10) [26]; Sekhri et al. (23) [56]; Whyle and Olivier (9) [23].
**Cluster 3 – blue**	Abuduxike and Aljunid (9) [57]; Barker et al. (7) [58]; Bottazzi et al. (15) [59]; Bottazzi and Brown (10) [60]; Hendriks et al. (6) [61]; Hotez and Ferris (119) [62]; Mahoney (18) [63].
**Cluster 4 – yellow**	Lambert et al. (18) [64]; Lei et al. (13) [65]; Naqvi et al. (18) [66]; Newell et al. (14) [67]; Saw et al. (10) [68]; Tin et al. (14) [69].
**Cluster 5 – violet**	Kruk et al. (14) [70]; Mwisongo and Nabyonga-Orem (9) [71]; Rao et al. (12) [72]; Saxenian et al. (23) [71].
**Cluster 6 - light blue**	Ali et al. (22) [73]; Basit et al. (7) [74]; Nishtar (10) [17]; Nishtar et al. (17) [75].
**Cluster 7 – orange**	Meredith et al. (23) [76]; Zhang et al. (39) [77]; Zhou et al. (12) [78].
**Cluster 8 – brown**	Alemnji et al. (22) [79]; Alemnji et al. (25) [80].

* The colour of each cluster is the same as that in the relative “bubble” shown in Fig. 4

**Table 3:** The quali-quantitative studies included in the sample

Scientific publications	Topic	Type of partnership	Main results
Molyneux & Zagaria [47]	Programme to eliminate lymphatic filariasis	Collaboration between LMICs, non-governmental development organisations and civil society organisations	The programme has expanded rapidly, with the annual number of people treated rising from 2.9 million (in 12 countries) in the year 2000 to 25.89 million (in 22 countries) in 2001
Peters & Phillips [48]	Mectizan donation program	Collaboration between international partners	The results of a survey of 25 partners show that the perceived benefits far outweigh the problems, and that the direct costs to the organisations have been minimal
Lambert et al. [64]	National Tuberculosis Programme in Bolivia	Collaboration between Bolivian government and private pharmacies	The first phase of the intervention proved effective in reducing the availability of the main tuberculosis drugs in pharmacies, and in improving referral of clients seeking tuberculosis drugs.
Saw et al. [68]	PPP to improve tuberculosis control in Myanmar	Cooperation between Myanmar Ministry of Health and private general practitioners	A considerable delay was found between the onset of symptoms of tuberculosis and seeking treatment. Old patients influenced the treatment seeking behaviour and choice of treatment clinics of new patients
Saxenian et al. [81]	Fiscal space analysis to calibrate appropriate levels of public financing for the new vaccines	PPP of LMICs, finance organisations, foundations, and the pharmaceutical industry	For LMICs, external financing will be required to purchase vaccines supported by Global Alliance for Vaccines and Immunization, so cofinancing needs to be modest
Ali et al. [73]	Emergency medical services in Pakistan	Collaboration between public administrations, non-governmental organisations, and the private sector	Systems analysis showed community participation to explain the project’s strength. Since its establishment, the project has been meeting its own recurrent expenditures without levying an extra burden on the government

The type of collaboration, the roles of the different partners and the objectives pursued by the partnership were used as key elements to assign each article to the relevant RA. The process of analyzing the articles led researchers to identify four main areas of research. Table 4 shows the distribution of 47 articles over the various RAs.

**Table 4:** PPP in LMICs: research areas, sections and scientific publications

Research Area	Section	Scientific publications
**Transfer of resources**	*Tangibles resources*	[16, 47, 50, 57, 61, 71, 72, 81]
	*Intangibles resources*	[43, 44, 48, 74, 76]
**Co-production of health goods and services**	*Contract-based agreement*	[30, 53, 54, 56]
	*Non-contractual based agreement*	[51, 58–60, 62, 73, 80]
**Governance networks approach**	*Transnational partners*	[17, 25, 49, 76, 45, 46; 69, 70, 78, 79]
	*Local partners*	[26, 64, 66, 68, 79]
**Criteria for successful partnership development**	*General framework*	[2, 23].
	*Specific issues*	[5, 52, 55, 63, 65, 67]

### RA 1: transfer of resources

The transfer of resources between actors in the same partnership is used as the criterion for including articles in RA 1. Buse and Walt have defined Global Public-Private Partnerships (GPPP) as a “collaborative relationship that transcends national boundaries” [16]. Facilitation of relationships between international and local partners is a key WHO role and Buse and Walt find WHO involvement in nine out of thirteen GPPP programs [16]. Studies by Anderson [43], Gupta et al. [44], Peters and Phillips [48], Meredith et al. [76], Hendriks et al. [61], and Basit et al. [74], demonstrate this involvement.

To identify RA 1 more clearly, a further classification was made, into those dealing with tangible and those dealing with intangible resources (Table 4). Buse and Walt make a classification of GPPPs into three categories: product-based, product development-based and systems-based [16]. Other authors focus on individual international drug transfer programs for the control of specific diseases, such as lymphatic filariasis [47], onchocerciasis [48, 76], and HIV/AIDS [72]. Equally interesting are the studies by Abuduxike and Aljunid [57] and Hendriks et al. [61] which describe technology transfer projects for the production of drugs for developing countries. Saxenian et al. report the case of the PPP created to help the poorest countries introduced new vaccines thanks to joint financing by other international partners [81]. Finally, Mwisongo and Nabyonga-Orem offer a literature review on GPPP highlighting the persistent challenges in this field [71].

The literature identifies PPPs transferring intangible resources as those working with experience, know-how and skills. Pfizer’s Global Health Fellows Program aims to promote better health by improving the service delivery capacity of local partners in poor countries [50]. Basit et al. describe several training projects for the transfer of know-how for diabetes monitoring and surveillance from western countries to Pakistan [74]. Gupta et al. describe the Green Light Committee as a multi-institutional health-base partnership with the aim of making recommendations for the control of tuberculosis [44]. Lastly, Anderson presents a PPP for the transfer of appropriate expertise for the treatment of tobacco dependence from high- to LMICs [43].

### RA 2: co-production of health goods and services

Academic articles describing PPP developed for co-producing health goods and services in LMIC health systems fall under RA 2. Here it is possible to classify two groups of articles using type of PPP (Table 4). The first group includes partnerships in which a public service is funded by a formal partnership between the government and the private sector. These PPPs involve a low number of actors, and show a clear separation of roles. The government is final payer of healthcare while the private partner is responsible for co-financing, maintaining and delivering services. In health PPP, the contractual agreement creates a level of accountability in cost management and quality that may be difficult to achieve it if the government is both the purchaser and the provider of care [30].

The second group in RA 2 contains the most articles with no clear distinction of the roles between different actors. However, unlike GPPPs, PPPs do not transfer resources, rather there is a sharing of resources to achieve a common goal. Some authors recognise such PPP as Product Development PPP [60, 62, 77]. These studies describe strong collaboration projects to develop drugs and vaccines against neglected tropical diseases [80], and provide healthcare service [73]. The advantage is that each actor contributes towards the achievement of a broader goal which a single organisation would be unable to achieve.

### RA 3: governance networks

RA 3 includes articles about governance networks. Governance Networks can be found in types of PPP in which governments run schemes based on the involvement of different stakeholders in developing strategies and making decisions [82]. The health sector is one of the most complex sectors to govern and manage and, LMICs often suffer of weak capacity to perform regulatory functions [26]. Private actors often have large resources available, as well as the power to obstruct policy interventions, and it can be the case that only through collaborative action can health issues be solved [82]. Examples of dialogue between LMIC governments and private organisations have multiplied, along with attempts to involve private partners in strategic planning for the health sector [26]. Articles included in this RA can be classified into two groups (Table 4). The first group includes Governance Networks made up of transnational and local partners, and the second group includes government and local partner networks. Hein and Kohlmorgen write that a transnational Governance Network is needed to face global health issues [45]. They find that although WHO is the main global health organisation, the World Bank is the biggest donor and should thus have a voice with WHO on global health policies. Further examples are identified in studies addressing different contexts, such as maternal health in Africa [46, 70]; malaria and tuberculosis [49] neglected tropical diseases [77] in sub-Saharan Africa [49], non-communicable diseases in Pakistan [17, 75], infections in Eastern Asia [78] and vaccines in Myanmar [69]. These studies emphasise the importance of conceiving of international institutions as strategic players involved in the definition of health policies and programs.

The articles in the second group describe partnership between the public and private sectors where government is not solely responsible for delivering health policies and programs, but can rely on local organisations with complementary mandates. Alemnji et al. recommend that governments in developing countries should organise committees involving private stakeholders to develop standard manuals and policies relating to HIV diagnosis [79]. Palmer [26] describes efforts made in six LMICs in establishing consultative forums for the public and private sector to define shared health programs. In the study by Lambert et al., the Bolivian government collaborates with local private pharmacies for tuberculosis control [64]. Finally, both in Pakistan [66] and in Myanmar [68], the government and the private partners have collaborated to develop a national tuberculosis program.

### RA 4: criteria for successful partnership development

RA 4 includes eight articles about building a successful PPP. Since PPPs have become a common approach to health problems in LMICs, there is an increasing need in literature to identify the conditions which make a PPP effective. Buse and Waxman study the potential risks and benefits of PPPs, and recommended that before investing in the PPP model, governments closely investigate good partnership practices and how to leverage the private sector contribution to health development [83].

The articles in RA 4 are grouped into two sections (Table 4). The first group aims to isolate the main characteristics of successful PPP building in general. Barr, identifying eight principal aspects, develops a protocol to evaluate the effectiveness of PPPs [2]. Whyle and Olivier classify partnerships into eight categories and identify characteristics and critical success factors for each of them [23].

The remaining six articles identify the criteria for a successful partnership focusing on specific issues. Johnston and Finegood find three key factors for building a successful PPP to fight obesity and non-communicable diseases [5]. The studies by Newell et al. [67] and Lei et al. [65] both focus on tuberculosis. Newell et al. identify characteristics of an effective PPP in two areas: leadership and management issues, and technical issues [67]. Lei et al. identify as crucial for PPP the areas of financial costs, governance, communications and trust [65]. The last three articles have different focuses. Mahoney proposes six determinants for PPP in health technology innovation [63]. Ejaz et al. suggest how to have successful collaboration with non-governmental organisations [52]. Finally, Palmer and Mills focus on PPP contracts examining which elements influence the nature of the contractual relationship [55].

## Discussion

### Implication 1. Topics, timing and author contributions to the debate

This article builds on analysing a sample of 148 articles published during the timeframe 1993–2018. As noted above, one article was published in 1993 and no citations were received for twenty-five years. The second one came in 1998, and from then on research has continued. A first spike in the number of published items from 2011 onwards occurred in 2016 when 23 articles were published. Development of research has been fragmentary; many articles stand alone and no one researcher or research group emerges as working particularly in this field over the timeframe. This yields the insight that there is no “superstar effect” [39], an effect which occurs when there is extreme specialisation of a small number of influential authors delivering the majority of studies.

### Implication 2. Journal specialisations and impact

The first consideration concerning journal specialisation is that although the most influential journals in terms of citations received are ranked in this SLR, there are in fact no journals published with a specific focus on the field, and authors in this field appear to have no preference for any specific publications. However, ranked by number of items and citations, the two top journals dealing with general health issues are *Bulletin of the World Health Organization* and *Tropical Medicine and International Health*, and the top three focussing on health management and economics are *Health Affairs*, *Health Research Policy and Systems* and *Health Policy and Planning*. A useful recommendation for researchers in this field is thus to consider both general health and specialised health management and economics journals.

### Implication 3. Future research agenda and policy recommendations

Analysing the 47 scientific articles, four main RAs are identified. Each RA supplies suggestions for a future research agenda, and managerial and policy implications for PPP in LMICs.

RA 1 includes articles about GPPP. In many LMICs, health services are provided in poor facilities characterised by frequent shortages of medicines and supplies. The transfer of resources through GPPP from western countries to LMICs is a common response to such problems. The focus has shifted away from GPPP studies on drug donations by developed to developing countries, towards new forms of partnership more oriented to the technology transfer for the production of drugs and vaccines directly in LMICs [57]. RA 1 findings are useful for LMIC managers and policy-makers. In the contexts of enormous health problems, local governments are required to take the first steps toward engaging national and international partners to meet the demand for key public health priorities. Lack of access to technical knowledge is the main constraint for LMIC manufacturers. It is therefore important to support knowledge transfer through training curricula, courses and technology transfer programs.

RA 2 focuses on PPPs for the co-production of health goods and services. Improvements in LMIC health systems require approaches that should simultaneously address not only infrastructure and financing, but also access and management, to achieve better patient outcomes [56]. Most studies in RA 2 show that sharing public and private resources can bring benefits in terms of efficiency, equity, and cost reduction. In fact, one of the main reasons for developing a PPP is economic. When private partners are involved, pressure on the public purse declines and allocation of economic resources becomes more efficient [84]. And from their point of view, PPP allows private companies to enter new markets, find new investment opportunities, reduce long-term uncertainties, share risks, and access public grants [85].

However, although PPPs offer robust potential for service delivery, they cannot be considered as a panacea for resolving every health issue. The failure of some PPP experiences suggests that a PPP is not always an appropriate solution [67]. In general, there is no clear strong evidence in literature showing that a PPP approach is preferable to more traditional models [2]. The critical question is how an underfunded government can co-operate with private organisations which traditionally benefit from external sources of funding and which are technically and financially stronger [26]. One of the major criticisms of PPP is that the private sector has several mechanisms for maximising profits which may conflict with the goal of better public health. Governments, perhaps because a greater alignment of motivation and shared goals, generally find the prospect of managing a relationship with non-profit organisations less daunting.

The private sector can be also an important player for LMIC health systems in defining policies and programs (RA 3). In several LMICs, gaps in the regulatory framework were noticeable. The inability to perform the regulatory function can lead to an erosion of trust in the state as manager and provider of healthcare, and such breakdown of trust can in turn cause the worsening of public health services [49]. One way of reinforcing the role of health policy-makers is through the engagement of private actors in the decisional processes [83]. This idea is based on the awareness that health issues involve multi-level, multi-actor, and multi-sectoral-challenges and cannot be solved by a single actor. A Governance Network is a new perspective of governance where policy is horizontally influenced by private organisations and/or civil society actors [86]. Most articles are included in this RA, nevertheless, further studies are required for at least two reasons. First, the engagement of private actors in the definition of health policies is recognised as a new frontier in LMIC health issues [87]. Second, many critical issues including governance structure, network accountability, are still underexplored [46, 70]. For all these reasons, governments should provide effective mechanisms for engaging external stakeholders in the national process, ensuring their participation in the formulation of health policy and implementation of national plans.

The last research area, RA 4, includes articles identifying criteria for a successful partnership. A common issue affecting every type of PPP is recognition of factors enabling a partnership to reach its own objectives. In RA 4, six of the eight articles focus on features for building an effective PPP starting from specific contexts or issues. Three of the articles focus on specific diseases [5, 65, 67], one on health technology innovation [63], one on PPP with non-governmental organisations [52], and one on contractual issues [55].

Although most articles in RA 4 focus on specific issues, they all identify two broad areas as crucial for building a successful partnership. The main potential risks include: combination of the organisation’s goals, the slowness of bureaucracy, conflict of interest, which can be destructive for weaker members. Secondly, differences in inter-organisational cultures, and difficulties in establishing appropriate means of measuring accountability and performance need to be addressed. In the light of these considerations, criteria for success are: the alignment of strategy; good governance practices; and strong project management with clear expectations of benefits, roles and responsibilities. Managers should be aware that in setting up a successful PPP, the initial stages can be difficult, largely due to suspicion among partners. Public actors play a leadership role in enhancing dialogue, and encouraging partners to believe in the partnership.

Although this study provides innovative “food for thought”, some limitations need to be addressed. The dataset used does not include the grey literature. This decision was taken in order to exclude preliminary studies in fields which are at the beginning of their development. This dataset also excludes articles not indexed in the Scopus or Web of Science databases. A small number of false negatives can be expected during the selection process. Secondly, the paper does not provide information on why the evolution occurred over time, or details on the choice of journals used.

## Conclusion

Shortages of human, financial and material resources, which jeopardise the provision of quality health services, are still a serious challenge in LMICs [24, 26]. Saw et al. identify the circumstances which support the proliferation of PPP in developing countries as: political and economic changes, increasing demand for public quality services, and health sector reforms [68]. However, PPPs vary in structure, partners, and objectives, as well as in the results achieved. This SLR has clarified these aspects and should facilitate future studies on how the functioning of PPP can be improved.

The classification into four RAs offers additional implications in terms of continuous updating and longitudinal investigations for each area of investigation. Few studies evaluating the impact of PPP on clinical performance outcome have been found [30, 53, 54], and there is a clear need for studies monitoring long-term clinical outcomes. Finally, in aiming to improve health systems, managers and policy-makers often need to decide whether it is riskier to continue to pursue what has not worked in the past, or to try new approaches [56].

## Supplementary information

**Additional file 1: PRISMA Checklist**. PRISMA 2009 checklist for the systematic literature review on public-private partnership in low- and middle-income countries.

## Data Availability

The datasets analysed during the current study are available from the corresponding author on reasonable request.

## Abbreviations

LMIC: Low- middle-income country
WHO: World Health Organization
PPP: Public-private partnership
SLR: Systematic literature review
RA: Research area
CPY: Citation per-year index
GPPP: Global public-private partnership
